# Socioeconomic status impacts Chinese late adolescents’ internalizing problems: risk role of psychological insecurity and cognitive fusion

**DOI:** 10.1186/s12889-025-22628-0

**Published:** 2025-04-16

**Authors:** Zhiyuan Tao, Zhenhai Wang, Yilin Lan, Wei Zhang, Boyu Qiu

**Affiliations:** 1 School of Health Management, Guangzhou Medical University, Guangzhou, Guangdong 511436 China; 2 School of Psychology, South China Normal University, Guangzhou, 510631 China; 3 Foshan No. 1 Middle School, Foshan, 528099 China

**Keywords:** Socioeconomic status, Late adolescents, Internalizing problems, Psychological insecurity, Cognitive fusion

## Abstract

**Introduction:**

Low socioeconomic status (SES) has been shown to be associated with more internalizing problems, but the mechanism of this relationship has not been investigated in Chinese late adolescents.

**Methods:**

High school students (*N* = 780, 54.45% girls) were recruited to complete questionnaires to assess SES, anxiety, depression, and cognitive fusion. We tested the hypotheses that this association would be mediated by the psychological insecurity and moderated by their cognitive fusion.

**Results:**

Regression-based analyses indicated that (1) psychological insecurity fully mediated the relationship between SES and internalizing problems; (2) late adolescents with high vs. low cognitive fusion showed a stronger positive association between psychological insecurity and internalizing problems.

**Discussion:**

The results highlight the important effects of psychological security and family resources on late adolescents’ mental health and give implications for interventions aimed to reduce adolescent internalizing problems through acceptance and commitment therapy.

## Introduction

Many countries were successful in reducing poverty in the last few decades [1], but the poverty rate has increased since the inception of COVID- 19. Adolescents living in poverty are exposed to multiple risk factors, such as a suboptimal living environment (e.g., poor housing, community violence, food insecurity) and psychosocial stress (e.g., family conflict, peer discrimination). Low income and low parent education are two common contributors to poverty [2], and living in poverty early in life has been shown to predict developmental trajectories of physical, externalizing, and cognitive disorders [3–5]. Youth from families with low-socioeconomic status (SES) may show defects in brain development [6], including brain structure and functions that subserve executive function and emotion regulation [7].

In addition to the above problems, there appears to be a link between poverty and internalizing problems. The term internalizing was first proposed by [8] to describe psychopathological spectrums of developmental problems. Internalizing problems refer to a continuum of symptoms of excessive control or excessive concern for the inner self (e.g. anxiety, depression, social withdrawal, and physical discomfort) [9]. Among them, anxiety and depression have had a high prevalence in recent years [10]. These problems have been shown to have a negative impact on public health and place youth at risk for future mental health problems (e.g., suicide attempts and bipolar mood disorder) [11]. Poverty is one risk factor for youth internalizing problems, and the relationship between the two variables is robust among children and adolescents [12]. A meta-analysis including 120 effects showed that SES was significantly, negatively associated with mental health problems in adolescents [13].

The family investment model and the family stress model could be useful to explain this relationship. The family investment model maintains that parents with low SES face difficulties in affording adequate housing in safe communities, and their limited time and energy create challenges in accompanying their children [14]. These limitations in resources may increase the risk of children’s psychological problems [13]. In addition, the family stress model maintains that lower income may put stress on parents and thus put families in a turbulent state that leads to children’s emotional problems [14]. Family economic stress increased the unpredictability of family environments and the risks to youth in low-SES families [15]. Both theoretical considerations and empirical evidence suggest that there is a negative relationship between SES and youth internalizing problems. In China, where the current study was conducted, there has been a wide promotion of the policy of precise poverty alleviation (i.e., giving economic help and agricultural technology support, and improving basic production and living conditions in poor areas) [16]. The concept of relative poverty has been paid more and more attention which means that there is a need to explore the impact of low family SES on youth development. Identifying the mechanism by which SES affects internalizing problems can help us further understand this relationship and provide suggestions for interventions.

### Mediating mechanism of psychological insecurity

Previous interpretations of psychological insecurity were not uniform [17, 18]. Researchers typically view psychological insecurity as interpersonal or emotional insecurity and may ignore the measurement structure of psychological insecurity [17]. We believe that the concept of psychological insecurity should include not only the sense of feelings of rejection and the anxiety of isolation but also the feelings of uncertainty and not being able to control the environment [19, 20].

Highly psychologically insecure adolescents might diligently chase interpersonal security and a sense of emotional safety, a primary goal of adolescents [21, 22]. However, adolescents in low-SES families may suffer more family conflict, which blocks them from perceiving positive emotions and safety [21]. Compared to children and early adolescent, late adolescents are more able to capture and interpret the threat posed to their security by economical stress and interparental conflict. At the same time, adolescents in low-income urban areas are more likely to suffer from chronic environmental stress, such as community violence [23]. Such an unpredictable environment decreases adolescents’ sense of control over the environment and then increases their psychological insecurity.

Adolescents with high psychological insecurity may view neutral cues as aggressive and threatening, and therefore they may perceive people and environments as full of danger [21, 24]. Moreover, psychologically insecure adolescents may assume that negative events are always with them, and these events are uncontrollable [25]. Therefore, adolescents with low psychological security may not focus on solving problems but instead stay in a state of sadness [12]. This group may report higher rumination, a behavior that can increase depressive symptoms [26]. Insecure adolescents may enter a state of being ready to deal with interpersonal and environmental risks at any time, contributing to their depression and anxiety. Given that low SES might be associated with higher interpersonal insecurity and a sense of the environment as uncontrollable, psychological insecurity could be a mediator of the association between a stressful environment and psychological maladjustment [27].

In addition, the assessment of insecurity of the environment was amplified during the pandemic; if individuals in low SES families got infected, they may not have enough financial resources to tide them over [28]. Meanwhile, parents’ unemployment and other social system barriers may cause adolescents to predict future turbulence in the family’s economic situation, resulting in a greater sense of psychological insecurity [29]. Adolescents would try to preserve their psychological security in a risky environment, which requires a large expenditure of psychological resources and limits their development [30], such expenditure may be higher than normal period. A study in developing countries has already shown that individuals who felt psychologically insecure reported higher levels of anxiety and depression [31]. Therefore, we believe that it is necessary to consider psychological insecurity as a mediator in the relationship between low SES and late adolescents’ internalizing problems.

### Cognitive fusion as a potential moderator

As the third wave of cognitive-behavioral therapy, Acceptance and Commitment Therapy (ACT) [32] regards psychological inflexibility as a key mechanism of psychopathology and regards enhanced psychological flexibility as the central goal of treatment [33, 34]. ACT has been proven to be widely effective in intervening with various mental health issues in adolescents, such as anxiety, depression, trichotillomania, and substance abuse, and it can significantly improve adolescents’ well-being [35–37]. Cognitive fusion is one of the key components of psychological inflexibility in the structure of ACT. It is described as a psychological phenomenon in which one’s emotions and behaviors are inflexibly regulated and dominated by the literal meaning of one’s thoughts [38]. In other words, people with cognitive fusion tend to consider their thoughts as irrevocable truths and then act in accordance with such thoughts regardless of the current, real context.

It can be expected that people who show inflexibility in the form of cognitive fusion may experience emotional distress when thoughts automatically trigger other thoughts [33]. This possibility is consistent with the assumption that cognitive fusion would affect multiple domains of mental health. People with high cognitive fusion may express emotional distress without the existence of actual painful events. Previous studies have consistently shown that cognitive fusion is positively associated with internalizing problems [39, 40], depression and anxiety [41], and alexithymia [42].

Specifically, cognitive fusion may exacerbate the impact of insecure cognition (e.g. always worrying about something bad happening) on adolescent mental health [43, 44]. Individuals with high cognitive fusion may amplify unstable and unpredictable thoughts about their environment, becoming trapped in them. This makes them more prone to persistent worry about their surroundings, ultimately triggering anxiety. Cognitive fusion may also lead people to obstinately act in accordance with their intrusive thoughts [45], Individuals whose behaviors are closely tied to thoughts about environmental and interpersonal risks may opt out of activities to avoid potential unmanageable risks and the possibility of interpersonal rejection. This could increase their absolutist view that something bad will happen, leading to more internalizing problems. In contrast, people who do not show cognitive fusion would test their thoughts against actual situations. For example, after repeatedly experiencing psychological security in interactions with others, their psychological insecurity will be challenged and then weaken and even disappear. Additionally, adolescents with low psychological safety tend to exhibit higher interpersonal avoidance and lower help-seeking behaviors. High levels of cognitive fusion may further exacerbate these negative interpersonal patterns. They may believe that all problems must be handled independently, abandoning social support, which in turn increases their likelihood of experiencing anxiety and depressive emotions. Therefore, by using ACT to cultivate an individual’s psychological flexibility, it is possible to counteract psychological insecurity, reduce negative emotions and inner conflicts in adolescents, and lessen their uncertainty when facing unknown environments, thereby alleviating their internalized issues [46, 47].

All in all, adolescents with high cognitive fusion would fuse more insecure thoughts with their emotions and behavior. We suggest that excessive fusion with thoughts of overestimating interpersonal and environmental insecurity may be an important moderating factor that exacerbates the relationship between psychological insecurity and internalizing problems. However, no study to date has tested this inference. In the current study, we tested the moderating effect of cognitive fusion on the association between low SES adolescents’ psychological insecurity and internalizing problems.

### The present study

**Fig. 1:**
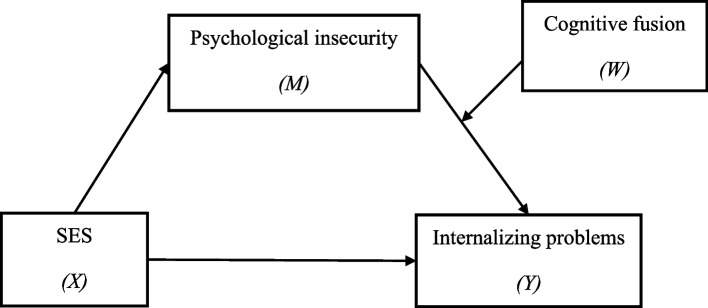
The proposed moderated mediation model

The present study was guided by ACT and used a model of moderated mediation to test the following hypotheses (Fig. 1).

- *Hypothesis 1*: SES is significantly related to greater adolescent internalizing problems.
- *Hypothesis 2*: Psychological security mediates the association between SES and adolescent internalizing problems. Specifically, SES is correlated with less psychological insecurity, while psychological insecurity is correlated with more internalizing problems.
- *Hypothesis 3*: Cognitive fusion moderates the pathway from psychological insecurity to adolescent internalizing problems. Specifically, the relationship between psychological insecurity with internalizing problems would be stronger among adolescents with high cognitive fusion.

To validate the theoretical model, this study used a convenience sampling method to recruit all 11 th-grade students from a high school. Participants were asked to answer a series of questions related to their family’s socioeconomic status and complete three self-report scales: the Anxious/Depression subscales of the Youth Self-Report, the Security Questionnaire, and the Cognitive Fusion Questionnaire. This helps theoretically explain how socioeconomic status influences high school students’ mental health through the sense of security in community samples. Practically, the exploration of the moderating role of cognitive fusion provides insights into how ACT could serve as a potentially effective intervention.

## Method

### Participants

Participants were recruited from high schools (grades 11) in a second-tier city in southern China by convenience sampling in December 2020. The survey was conducted during a period of sporadic COVID- 19 outbreaks. Using a convenience sampling method, we directly surveyed all 11 th-grade students at the school. A total of 808 late adolescents (54.45% females, *n* = 440) ranging in age from 16 to 19 (*M*_*age*_ = 16.81, *SD* = 1.00) participated in this study, and 28 subjects (18 girls) were excluded according to the polygraph items, 780 data were used in the analysis. According to review articles on sample size requirements for questionnaire-based studies, constructing a moderated mediation model commonly requires 300 valid responses. In questionnaire-based pathway statistical models, each estimated parameter typically requires a sample size of 20. In our study, the four-variable path model, under the condition of including gender and age as control variables, contains 9 parameters that need to be directly estimated, so at least 180 valid responses are required. The data included in this study meet this criterion, ensuring that the subsequent model results are valid and reliable [48]. By using convenience sampling for an entire grade, we were able to obtain a sample size exceeding what is required to construct the theoretical model, ensuring the stability of the study’s results.

### Measures

#### SES

In the field of poverty psychology, income and parental education are usually measured simultaneously to observe the complex effect of poverty. We measured the per capita income of the family, rated from 1 (0–1000 ¥) to 10 (more than 9000 ¥); and father and mother education, rated from 1 (primary school or below) to 6 (master or higher); and home location, rated from 1 (rural area) to 5 (city center). We standardized the results of four items to eliminate the influence of units, then summed the four Z-scores to calculate the SES score. According to the 2021 review of per capita income in the city where the sample was collected, the average per capita income in 2020 was approximately 4,687 RMB. In our study, the reported average per capita income was 5.85 (approximately 5,850 RMB). The proportion of participants reporting incomes below 5,000 RMB was 46.8%, and those below 2,000 RMB accounted for 9.7%. This indicates that while the sample income is slightly above the city average, it still includes lower-income groups, ensuring representativeness.

#### Internalizing problems

Internalizing problems were measured by the Anxious/Depression subscales of the Youth Self-Report [49]. The questionnaire consists of 16 items assessing negative emotions during the past 6 months, such as “unhappy and sad” “nervous and tense” and “lonely”. Each item is rated from 0 (not true) to 2 (very true), with a higher mean score indicating a higher internalizing level. The YSR has been used to measure internalizing problems and showed great reliability and validity in a previous Chinese study [50]. In the current study, Cronbach’s α coefficient of the scale was .86.

#### Psychological insecurity

The Chinese version of the Security Questionnaire [51] was used to measure psychological insecurity. This questionnaire consists of 16 items. Half the items assess interpersonal security (e.g., “I never dared to say what I thought”) and the other half of the items assess certainty in control (e.g., “I feel that life is full of uncertainty and unpredictability”). Items are rated on a 5-point scale ranging from 1 (not at all true) to 5 (always true) and the means of all items were calculated, with a higher score indicating higher psychological insecurity. This questionnaire has shown good reliability and validity in previous Chinese studies [51]. In the current study, Cronbach’s α coefficient of the scale was .87.

#### Cognitive fusion

The Cognitive Fusion Questionnaire was used to assess adolescent cognitive fusion [38]. The questionnaire consists of 9 items rated from 1 (very inconsistent) to 7 (very consistent), the mean score of all items was calculated, the higher scores, the higher cognitive fusion. Example questions include, “Some ideas make me worry and pain” and “I was troubled by some ideas and could not complete the tasks”. The questionnaire has shown good reliability and validity in Chinese children and adolescent samples [52]. In the current study, Cronbach’s α coefficient of the scale was .91.

#### Procedure

This research was conducted with permission from the Academic Ethics Review at the university with which the first author is affiliated. It was approved by the Ethics Committee of the Department of Psychology, South China Normal University (protocol code: SCNU-PSY-2021 - 098). We have determined that all the measurement and research processes are observed by the Declaration of Helsinki. Before beginning the investigation, parents provided informed consent for their adolescents to participate. At the beginning of the data collection session, participants emphasized their answers to the questionnaires would be anonymous, questionnaires would seal up for safekeeping immediately, and only used for academic research. Adolescents were then instructed to complete the questionnaires independently. Data collection took place in the students’ regular classrooms during regular class time and finished in 30 minutes.

#### Statistical analysis

Data analysis was conducted with SPSS 26.0 in three steps. Firstly, Harman’s single-factor test was used to assess the possibility of common method bias and found that 32.76% of the variance could be explained by the first principal factor, which is less than 40% [53] indicating that no common method variance was detected. Secondly, descriptive statistics and Pearson’s correlation analysis were calculated. Finally, SPSS macro PROCESS 3.5, Model 14, with a 95% bias-corrected confidence interval (CI) based on 5000 bootstrap samples was used to examine the mediator and moderator in the models.

## Results

### Preliminary analyses

Table 1 displays the means, standard deviations, and correlation coefficients for all variables. The results showed that SES was negatively correlated with psychological insecurity. Moreover, psychological insecurity, internalizing problems, and cognitive fusion were positively correlated with each other. Although no significant correlation was found between SES and internalizing problems, this does not rule out the possibility of a mediating pathway. Therefore, we conducted a follow-up path model analysis. Gender and age were controlled in further statistical analyses.

**Table 1:** Descriptive statistics and correlations for all variables

Variables	1	2	3	4	5	6
1.Gender	-					
2.Age	-.05	-				
3.SES	-.04	-.03	-			
4.PI	.12**	.03	-.12**	-		
5.IP	.17***	.00	-.05	.67***	-	
6.CF	.14***	-.00	-.02	.48***	.55***	-
*Mean*	1.54	16.81	0	41.38	24.61	43.06
*SD*	0.50	1.01	2.83	11.50	6.41	13.87

*N* = 780, gender was dummy coded such that 1 = male, 2 = female

*SES* social economic status, *PI* psychological insecurity, *IP* internalizing problem, *CF* cognitive fusion

^**^p <.01

^***^ *p* <.001

### Testing for moderated mediation

All variables were standardized before being included in the model to eliminate the influence of different units. Model’s results showed that SES was negatively related to psychological insecurity (*β* = -.04, *t* = − 3.36, *p* <.001, Table 2), and psychological insecurity was positively related to internalizing problems (*β* =.51, *t* = 18.50, *p* <.001), but the residual direct pathway of SES and internalizing problems was not significant (*β* =.01, *t* = 0.72, *p* =.47). Cognitive fusion had a significant positive correlation with internalizing problems (*β* =.35, *t* = 12.10, *p* <.001), and the moderating effect of cognitive fusion was significant because psychological insecurity and cognitive fusion had a significant positive interactive effect on internalizing problems (*β* =.16, *t* = 7.68, *p* <.001). Moreover, the bias-corrected percentile bootstrap results indicated that the indirect pathways between SES and internalizing problems via psychological insecurity were both significant in adolescents with low cognitive fusion (indirect effect =.01, *SE* =.00, 95% CI [-.03, -.00]) and high cognitive fusion (indirect effect =.03, *SE* =.01, 95% CI [-.05, -.01]). Therefore, the mediating pathway was moderated by cognitive fusion, and the moderated mediation model was established.

**Table 2:** Model test results of moderated mediation model

Outcome variables	Model 1 (PI)			Model 2 (IP)		
	*β*	*t*	95% CI	*β*	*t*	95% CI
***Moderated mediation model: CF as a moderator***						
Gender	.12^**^^*^	**3.32**	**.05,.19**	.08^**^	**3.23**	**.03,.13**
Age	.03	0.80	-.04,.10	-.01	− 0.36	-.06,.04
SES	-.04^**^^*^	**− 3.36**	**-.07, -.02**	.01	0.72	-.01,.02
PI	**-**	-	-	.51^***^	**18.50**	**.46,.57**
CF	-	-	-	.35^***^	**12.10**	**.29,.40**
PI × CF	-	-	-	.16^***^	**7.68**	**.12,.20**
***Indirect effect***				*β*	*SE*	95% CI
Indirect effect of (*Mean - 1SD*) of CF				**-.01**	**.00**	**-.03, -.01**
Indirect effect of (*Mean*) of CF				**-.02**	**.01**	**-.04, -.01**
Indirect effect of (*Mean + 1SD*) of CF				**-.03**	**.01**	**-.05, -.02**
***Power of model***						
*R*^*2*^	.03^***^			.55^***^		
*F*	**7.84**			**158.99**		

Bold numbers mean significant effect

*SES* social economic status, *PI* psychological insecurity, *IP* internalizing problem, *CF* cognitive fusion

^**^ *p* <.01

^***^ *p* <.001

In recent years, constructing moderated mediating models in cross-sectional data has led many to doubt that cross-sectional research could say much about true mediation. We tested an alternative model to further reduce this error. Because youth were unable to decide on their family SES, it was reasonable to set SES as an independent environmental factor, however, the order of psychological insecurity and the internalizing problems remained in doubt. We tested the mediation model of “SES - internalizing problems - psychological insecurity” and found that SES was not related to internalizing problems (*β* = -.02, *t* = − 1.33, *p* =.18) and the mediating model was not established (indirect effect = -.01, *SE* =.01, 95% CI [-.03,.00]). We suggest our model was reasonable and could be used to explain the relationship between SES, psychological insecurity, and internalizing problems.

**Fig. 2:**
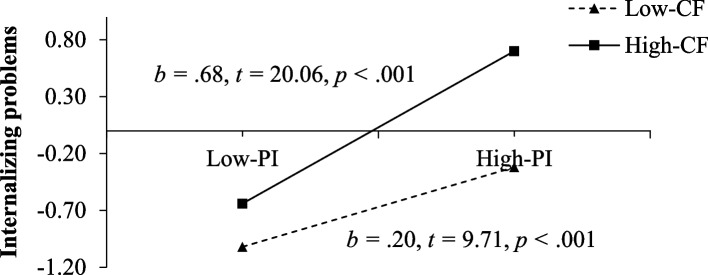
Simple slopes test of the moderating effect of cognitive fusion on the pathway of psychological insecurity to internalizing problems

Given that the moderating effect was significant, we conducted a simple slopes test to interpret the result. As shown in Fig. 2, the positive association between psychological insecurity and internalizing problems was significant among adolescents with low cognitive fusion (1 *SD* above the mean; *β* =.35, *t* = 9.71, *p* <.001) and high cognitive fusion (1 *SD* below the mean; *β* =.68, *t* = 20.06, *p* <.001).

## Discussion

China has made great strides in alleviating poverty [16], but there is nevertheless a need to understand Chinese families who live in low SES conditions. As of 2019, China’s urbanization has reached 60 %, and the cost of living in urban areas is higher than in rural areas [54]. In south China, where the current study was conducted, urbanization and economic development are even higher than in other areas of China, leading to higher prices. In other words, although our participants were not a poor group, the lower SES may have created difficulties for family members. Our results provide insight into the relationship between low SES and late adolescent internalizing problems.

Based on the literature, we assumed that late adolescents from low SES families would be at greater risk of internalizing problems. However, our results did not support Hypothesis 1. This may be because SES is one of the distal factors influencing late adolescent mental health development. Simply put, SES may directly affect late adolescents’ mental health or indirectly contribute to psychological risks through cumulative stress, damage to social support networks, and prolonged environmental pressures [55, 56]. Our findings support the latter perspective, suggesting that underlying economic stress, limited educational resources, less self-esteem and community instability associated with low SES influence adolescents’ perceptions of environmental and interpersonal safety. Then these insecurity thoughts may impact late adolescent mental health level. Additionally, our study was conducted in an urban setting, with sample families reporting slightly higher-than-average city incomes. These low-SES adolescents may not lack basic material security, which could explain why they do not directly perceive threats or experience strong negative emotions. The lack of a direct relationship between low SES and adolescent internalizing problems in our study suggests the need to test more complex associations with other factors.

Tests of mediation showed that SES was negatively related to late adolescent internalizing problems via psychological insecurity which provides support for our Hypothesis 2. These findings are important because other potential mediators of the effect of low SES on adolescent internalizing are environmental factors, such as community violence, that is hard to change. The discovery of a psychological mediating mechanism, namely psychological insecurity, provides insight into the impact of SES on internalizing problems at the psychological level. Psychological factors such as psychological insecurity and cognitive fusion are likely to be more amenable to change.

Parents with low SES face more difficulties in providing material resources and less time for positive parenting. Economic stress may also induce family conflict [14]. The previous study has provided support for these assertions, low SES is significantly associated with high interparental conflict, and these adolescents may report higher emotional insecurity [21]. Meanwhile, our sample was recruited in the pandemic period. Families with low SES are more likely to face even greater economic challenges during the COVID- 19 pandemic: families in low-income urban areas in developing countries may face the risk of unemployment and other social barriers, which may increase the health problems and economic insecurity of adolescents [28]. Based on these multiple perspectives, we conclude that low SES is indeed an important risk factor for adolescents’ psychological insecurity.

Further, psychologically insecure late adolescents may spend psychological resources to seek and maintain their psychological security in a threatening environment [30]. This excessive attention to the internal state may be associated with internalizing problems, and these problems may be further aggravated during the pandemic [31]. In addition, adolescents in low SES conditions may have negative cognitions, such as the belief that negative events in a dangerous environment are unavoidable [25]. Meanwhile, adolescents still depend on their families and are unable to change the current unsafe situation; thus, they may experience more helplessness and hopelessness [56].

Some empirical research also deliver indirect evidence of psychological insecurity as mediating the association between low SES and adolescents’ internalizing. Youth living in low SES families may tend to have a low threshold for judging situations as threatening, and this long-term insecure psychological state is related to higher adrenocortical reactivity [57]. This chronic state of psychological insecurity and ongoing stress reactions may make these adolescents more vulnerable when facing stress and may increase adolescents’ internalizing problems [57]. In addition, adolescents’ sensitivity to threats brought by high psychological insecurity may decrease the coupling of the amygdala and vmPFC, making adolescents less able to suppress their fear of threatening information [58]. In addition, the interaction between exposure to stress and coupling between the amygdala and vmPFC could predict later adolescent internalizing problems. Low SES also includes the potential insecurity of material conditions such as food and housing, and each type of material environment in parenting can independently lower adolescents’ mental health levels [59, 60]. We must also consider the potential risk of parental unemployment in the context of this study. All of these objective factors associated with low SES may be positively correlated with adolescents’ psychological insecurity, which in turn may be positively correlated with their psychological risk [61]. Combining our results with the past empirical evidence in many perspectives (e.g., neuroscience, biopsychology, nurturing material conditions), we believe that psychological insecurity is an important mediator in the association between low SES and adolescent internalizing problems.

Consistent with Hypothesis 3, cognitive fusion moderates the association between psychological insecurity and internalizing problems. Our study is pioneering as it is the first to investigate the interaction between cognitive fusion and psychological insecurity as a predictor of adolescent internalizing problems. Psychological insecurity is a maladaptive cognitive pattern that includes the perception that the outside world and other people are threatening, untrustworthy, and uncontrollable [62]. In addition, cognitive fusion makes psychological insecurity more harmful, as people with high cognitive fusion are more likely to fuse these insecure thoughts with seemingly uncontrollable actions based on these thoughts [63, 64]. In this state, there may be a reduction in psychological flexibility and the person may lose the ability to distance themselves from distressing thoughts and experiences [39]. These conditions contribute to the adolescent’s risk of developing internalizing problems.

In contrast, a key component of the ACT therapeutic framework is the reduction of cognitive fusion, which can be alleviated by the psychological intervention [65, 66]. For example, a randomized controlled trial on 243 adolescents found that after a 5-week web intervention based on ACT, participants showed a significant decrease in cognitive fusion and depression symptoms and showed a significant increase in psychological flexibility and well-being [67]. Therefore, for individuals experiencing psychological insecurity, interventions aimed at reducing cognitive fusion may be effective to prevent them from more internalizing problems.

However, it is worth noting that our results showed that even at a low level of cognitive fusion, psychological insecurity remained correlated with internalizing problems. Therefore, it is necessary to further intervene to improve the individual’s psychological security after the intervention is implemented to reduce cognitive fusion.

### Practical implications

In our sample, late adolescents’ mental health and well-being were challenged during the pandemic. In this context, the present study has several important practical implications. Our findings suggest that low SES is positively associated with internalizing problems via the mediating role of psychological insecurity. In addition, a previous study suggested that COVID- 19 has had a worse impact on mental health in groups with low SES [68]. Therefore, we propose that schools should provide more support and attention to adolescents with low SES if the environment is volatile to protect their psychological security. For example, suggest that school efforts to safeguard equitable access to digital connectivity, and provide emotional support to students, are beneficial to improve equity and inclusion, then increase the well-being of adolescents in low-SES families [69].

Our results also suggest that focusing on the psychological security of low-SES students is an important approach to prevent further internalizing problems. Though effective direct intervention strategies for psychological insecurity have not been conducted, evidence from recent studies could indirectly guide intervention. For example, a recent randomized controlled trial showed that an ACT-based online intervention program can reduce adolescents’ psychological insecurities related to future career development.

Most importantly, our results suggest that decreasing cognitive fusion in adolescents with high psychological insecurity may reduce the potential for further internalizing problems. Given that cognitive fusion is an important target in ACT, it is meaningful to consider how ACT could help adolescents in distress. Several studies have identified cognitive fusion as a target mechanism for addressing distress. We recommend emphasizing cognitive defusion to help low-SES adolescents break free from ineffective and excessive worries about family economic instability. By understanding that these subjective worries can’t solve real problems and may even prevent adolescents from noticing their parents’ emotional support. Additionally, everyone lives within a nested social system. Even in the face of family difficulties, adolescents can seek help from friends and teachers. Reducing interpersonal avoidance related to psychological insecurity can enhance social support and alleviate negative emotions. To reduce students’ psychological insecurity and cognitive fusion, educators can integrate ACT-based intervention into school psychology-related programs in the future.

### Limitations

The first limitation of this study is that our data were collected during the COVID- 19 pandemic. The youth in the study may have been experiencing higher psychological insecurity and greater internalizing problems than would have been seen before the epidemic in China. It remains to be seen whether the results can be generalized in the current social context. Secondly, under the framework of ACT, we tested cognitive fusion as a moderator, but there are other relevant moderators under this framework, such as committed action and acceptance, that could be tested. Thirdly, the result of Harman’s single factor test suggested that there may be a common factor linking the variables we tested. A non-adaptive cognition that “negative events cannot be avoided” behind psychological insecurity [25] may increase individual cognitive fusion and internalizing. Therefore, we suggest that future research should control the influence of factors such as non-adaptive cognition. Fourthly, the data were collected from a sample of late adolescents, and it is unclear whether the results could be extended to other age groups. A longitudinal and cross-lagged model is needed to confirm the relationships among SES, psychological insecurity, cognitive fusion, and internalizing problems. Finally, our assessment of internalizing problems cannot directly state the risk of depression and anxiety which need further detailed exploration.

## Conclusions

The results of this study in Chinese late adolescents showed that students from low SES families reported more psychological insecurity, and in turn, more internalizing problems. Adolescents with high cognitive fusion may fuse insecure thoughts and show a more robust relationship between psychological insecurity and internalizing problems.

## Data Availability

The datasets analyzed during the current study are available from the corresponding author upon reasonable request.
